# Retrograde pyelography before radical nephroureterectomy for upper tract urothelial carcinoma is associated with intravesical tumor recurrence

**DOI:** 10.1590/S1677-5538.IBJU.2019.0503

**Published:** 2020-07-31

**Authors:** Young Hwii Ko, Phil Hyun Song, Taeyong Park, Jae Young Choi

**Affiliations:** 1 Yeungnam University College of Medicine Department of Urology Daegu Republic of Korea Department of Urology, College of Medicine, Yeungnam University, Daegu, Republic of Korea; 2 Inje University Pusan Paik Hospital Department of Urology Busan Republic of Korea Department of Urology, Pusan Paik Hospital, Inje University, Busan, Republic of Korea

**Keywords:** Urography, Nephroureterectomy, Neoplasms

## Abstract

**Purpose:**

To investigate the association between preoperative retrograde pyelography (RGP), conducted to evaluate upper tract urothelial carcinoma (UTUC), and intravesical recurrence (IVR) after radical nephroureterectomy (RNU).

**Materials and Methods:**

Of 114 patients that underwent RNU, 72 patients without preoperative ureteroscopy and a history of bladder tumor were selectively enrolled. Variables associated with IVR were identified.

**Results:**

RGP was performed at a mean duration of 24.9 days prior to RNU in 41 (56.1%) of study subjects. During the mean follow-up period of 64.5 months, IVRs were identified in 32 (44.4%) patients at 22.3±18.8 (mean±SD) months after RNU. Despite similar tumor characteristics in the RGP and non-RGP groups, the incidence of IVR was considerably higher in the RGP group (63.4%) than in the non-RGP group (19.4%, p <0.001). The following variables differed significantly between the IVR and non-IVR groups: age (64.6±8.51 vs. 59.6±9.65 years), tumor location (lower or upper; 53.1% vs. 20%), tumor invasiveness (> pT2; 53.1% vs. 17.5%), preoperative hemoglobin (12.8±1.36 vs. 13.9±1.65), preoperative creatinine (1.29±0.32 vs. 1.11±0.22), and preoperative RGP (81.3% vs. 37.5%), respectively. Multivariate Cox regression model showed that tumor location (p=0.020, HR=2.742), preoperative creatinine level (p=0.004, HR=6.351), and preoperative RGP (p=0.045, HR=3.134) independently predicted IVR.

**Conclusion:**

Given the limitations of retrospective single-center series, performance of RGP before RNU was shown to have a negative effect on IVR after surgery.

## INTRODUCTION

Upper tract urothelial carcinoma (UTUC) accounts for 5-10% of urothelial neoplasms and 10% of renal tumors (1). Radical nephroureterectomy (RNU) with bladder cuffing is the established ‘gold standard’ for the management of UTUC (2). However, intravesical recurrence (IVR) occurs after 22-47% of procedures (3–5). Two dominant theories have been proposed to explain the mechanism of IVR: monoclonal and oligoclonal spread. According to the former hypothesis, IVR produces abnormal cell spread to the bladder before RNU or may increase the ability of locally budding tumors to release cancer cells into the urinary tract (6). On the other hand, the latter hypothesis involves carcinogenic exposure of the entire urothelial layer leading to independent multifocal tumor development within the urinary tract (7).

Increased rates of IVR after diagnostic ureterorenoscopy (URS) and prior to RNU have been reported by several authors, and a recent meta-analysis demonstrated an obvious association between the two (8, 9). These observations provide evidence that supports the monoclonal-spreading theory because URS with/without biopsy facilitates detachment and migration of abnormal urothelial cells into the lower urinary tract. For this study, we hypothesized that if URS truly increases the risk of IVR, then retrograde pyelography (RGP), which is a less invasive alternative to URS, might also give rise to IVR. In an attempt to identify the mechanism responsible, we investigated the effect of preoperative RGP on IVR after RNU among patients that did not receive preoperative URS.

## MATERIALS AND METHODS

### Patient collection and RGP procedure

Of 114 patients that underwent RNU for UTUC from January 2004 to June 2013 at our institution, 72 patients that did not undergo preoperative URS and had no history of bladder cancer were selectively enrolled, after approval obtained from the local institutional review board (YUMC 2017-12-018-001). Because our institution has no universal policy regarding the use of RGP as a radiologic tool to identify the presence of UTUC, the durations between RGP and RNU differed. However, RGP was performed as a separate procedure before RNU in all cases. RGP was performed by a urologic resident under local anesthesia using a 6Fr size ureteral catheter (open-end ureteral catheter, Cook Medical, Bloomington, IN, USA), which was inserted approximately 5-10cm from the ipsilateral ureteral orifice with cystoscope and fluoroscope guidance. At the time of RGP, the absence of suspicious bladder lesions was confirmed by cystoscopy. All 72 study subjects underwent computed tomography (CT) as a baseline diagnostic modality. At the time of RNU, bladder cuffing was performed by applying the open method, regardless of nephrectomy approaches (open or laparoscopic), using a modified Gibson incision.

### Clinicopathologic variables and follow-up after RNU

The histologic characteristics of UTUC were based on examinations of specimens obtained during RNU and included stage, grade, and lympho-vascular invasion (LVI). Tumor locations were determined by CT and RGP and dichotomized as upper (upper ureter and renal pelvis) or lower. Intra-ureteral tumor size was defined as the maximal tumor dimension within the urinary tract as determined by examining sagittal or coronal CT or RGP images. Hemoglobin and creatinine levels before RNU were also collected, due to their reported associations with IVR (6, 9, 10).

After RNU, patients were followed-up by four urological specialists who performed CT, cystoscopy, and urine cytology examinations on a 6- to 12-month basis for five years. Because of limited approval regarding the preventive use of intravesical chemo- or immunotherapy for UTUC in our country, no immediate intravesical therapy was implemented after RNU. When IVR was identified during follow-up surveillance, we recorded the duration between RNU and IVR.

### Statistical analysis

The endpoint of this study was the first IVR of urothelial carcinoma (UC) after RNU, which was defined as the first histologically confirmed bladder UC, regardless of tumor characteristics and number. The significances of differences between patients with or without IVR were compared by applying t and chi-squared tests, and associations between variables were identified using bivariate correlation analysis. Given the influence of time after RNU on IVR, the impacts of preoperative RGP and other clinicopathological factors on IVR were analyzed using a multivariate Cox proportional hazard model. The log-rank test was used for the comparison of each variable in a Kaplan-Meier model, and distribution normality was checked using the Kolmogorov-Smirnov test. The analyses were performed using SPSS version 21.0 (SPSS Inc., Chicago, IL, USA). Two-sided tests were used and the significance level was set at 5%.

## RESULTS

### Characteristics of the study subjects

Forty-one (56.1%) of the 72 study subjects underwent RGP to determine the presence of UTUC before RNU. Patient characteristics are summarized in Table-1. The 41 patients in the RGP group were significantly older than the patients in the non-RGP group (mean±SD; 64.5±8.9 vs. 58.3±9.0 years, p=0.005), but the results for the other variables (sex, LVI, preoperative creatinine, and hemoglobin levels, total operative time, and tumor size, location, stage, and grade) were similar in these two groups. Despite a shorter mean follow-up period (31.3±23.1 vs. 48.3±18.6 months, p=0.001), bladder tumor recurrence rate was markedly higher in the RGP group (63.4% [26/41] vs. 19.4% [6/31]; p <0.001). The mean time between RGP and RNU in the RGP group was 24.9 days.

**Table 1:** Characteristics of patients in the retrograde pyelography (RGP) and non-RGP groups.

		Total (N=72)			RGP (N=41)			No RGP (N=31)			P-value
		N	%	Mean (±SD)	N	%	Mean (±SD)	N	%	Mean (±SD)	
**Gender**	Female	14	19.4%		11	26.8%		3	9.7%		0.069
	Male	58	80.6%		30	73.2%		28	90.3%		
**Age (years)**				**61.81 (±9.44)**			**64.49 (±8.98)**			**58.26 (±8.99)**	**0.005**
	< 65	41	56.9%		16	39.0%		25	80.6%		<0.000
	≥ 65	31	43.1%		25	61.0%		6	19.4%		
**Length of tumor (mm)**				**34.92 (±16.3)**			**32.54 (±15.5)**			**38.06 (±17.0)**	**0.161**
	< 35	35	48.6%		25	61.0%		10	32.3%		0.016
	≥ 35	37	51.4%		16	39.0%		21	67.7%		
**Location of tumor**	Upper	47	65.3%		23	56.1%		24	77.4%		0.062
	Lower	25	34.7%		18	43.9%		7	22.6%		
**Multiplicity of tumor**	Single	67	93.1%		40	97.6%		27	87.1%		0.158
	Multiple	5	6.9%		1	2.4%		4	12.9%		
**T stage**	T1	48	66.7%		24	58.5%		24	77.4%		0.113
	T2	12	16.7%		7	17.1%		5	16.1%		
	T3	12	16.7%		10	24.4%		2	6.5%		
	Non-invasive	48	66.7%		24	58.5%		24	77.4%		0.092
	Invasive (≥pT2)	24	33.3%		17	41.5%		7	22.6%		
**Tumor grade**	low	24	33.3%		13	31.7%		11	35.5%		0.736
	high	48	66.7%		28	68.3%		20	64.5%		
**Lymphvascular invasion**	No invasion	64	88.9%		36	87.8%		28	90.3%		0.736
	With invasion	8	11.1%		5	12.2%		3	9.7%		
**Preoperative hemoglobin (g/dL)**				**13.37 (±1.61)**			**13.17 (±1.59)**			**13.65 (±1.61)**	**0.221**
	≥ 10	70	97.2%		39	95.1%		31	100%		0.212
	< 10	2	2.8%		2	4.9%		0	-		
**Preoperative creatinine (mg/dL)**				**1.19 (±.281)**			**1.22 (±.320)**			**1.14(±.217)**	**0.233**
	< 1.5	57	79.2%		29	70.7%		28	90.3%		0.043
	≥ 1.5	15	20.8%		12	29.3%		3	9.7%		
**Total operative time (minutes)**				**384.65 (±79.5)**			**368.78 (±95.7)**			**405.65 (±44.1)**	**0.051**
**Follow up period (months)**				**38.58 (±22.8)**			**31.27 (±23.1)**			**48.26 (±18.6)**	**0.001**
**Intravesical recur during follow up**	No recur	40	55.6%		15	36.6%		25	80.6%		<0.000
	Bladder recur	32	44.4%		26	63.4%		6	19.4%		
**Time from RGP to RNU (days)**				**24.93 (±19.9)**			**24.93 (±19.9)**				
	< 7	13	31.7%		13	31.7%		-			
	≥ 7	28	68.3%		28	68.3%		-			

### Variables associated with IVR

During the mean follow-up period of 64.5 months, 32 (44.4%) of the 72 study subjects developed IVR at a mean duration of 22.3±18.8 months after RNU. Table-2 summarizes differences detected between the IVR (n=32) and non-IVR (n=40) groups. Patients in the IVR group were significantly older (64.6±8.51 vs. 59.6±9.65 years; p=0.005). IVR group had a higher percentage of patients with lower tumor location (53.1% vs. 20%, p=0.032) and invasive tumors (> pT2; 53.1% vs. 17.5%, p=0.011). They had a lower preoperative hemoglobin level (12.8±1.36 vs. 13.9±1.65, p=0.004), a higher preoperative creatinine level (1.29±0.318 vs. 1. 11 ±0.221, p=0.009), and a higher percentage of patients underwent preoperative RGP (81.3% vs. 37.5%. p <001). However, the durations between RGP and RNU, which were normally distributed (two-tailed asymptomatic significance=0.531), were similar in these two groups (22.8±17.9 vs. 28.6±23.1 days, p=0.411). Tumor sizes, which were also normally distributed (mean 34.9mm; two-tailed asymptomatic significance=0.162), were also similar in the two groups (34.1±14.9 vs. 35.6±17.5mm, p=0.715).

**Table 2:** Characteristics of patients in the intravesical recurrence (IVR) and non-IVR groups.

		Total (N=72)			IVR (N=32)			No IVR (N=40)			P-value
		N	%	Mean (±SD)	N	%	Mean (±SD)	N	%	Mean (±SD)	
**Gender**	Female	14	19.4%		10	31.3%		4	10%		0.021
	Male	58	80.6%		22	68.8%		36	90%		
**Age (years)**				**61.81 (±9.44)**			**64.63 (±8.51)**			**59.55 (±9.65)**	**0.005**
	< 65	41	56.9%		13	40.6%		28	70%		0.012
	≥ 65	31	43.1%		19	59.4%		12	30%		
**Length of tumor (mm)**				34.92 (±16.3)			34.13 (±14.86)			35.55 (±17.49)	0.715
	< 35	35	48.6%		19	59.4%		16	40%		0.102
	≥ 35	37	51.4%		13	40.6%		24	60%		
**Location of tumor**	Upper	47	65.3%		15	46.9%		32	80%		0.032
	Lower	25	34.7%		17	53.1%		8	20%		
**Multiplicity of tumor**	Single	67	93.1%		32	100%		35	87.5%		0.061
	Multiple	5	6.9%		0	-		5	12.5%		
**T stage**	T1	48	66.7%		15	46.9%		33	82.5%		0.061
	T2	12	16.7%		8	25.0%		4	10.0%		
	T3	12	16.7%		9	28.1%		3	7.5%		
	Non-invasive	48	66.7%		15	46.9%		33	82.5%		0.011
	Invasive (>pT2)	24	33.3%		17	53.1%		7	17.5%		
**Tumor grade**	low	24	33.3%		7	21.9%		17	42.5%		0.065
	high	48	66.7%		25	78.1%		23	57.5%		
**Lymphvascular invasion**	No invasion	64	88.9%		28	87.5%		36	90%		0.737
	With invasion	8	11.1%		4	12.5%		4	10%		
**Preoperative hemoglobin**				**13.37 (±1.61)**			**12.79 (±1.36)**			**13.85 (±1.65)**	**0.004**
**(g/dL)**	≥ 10	70	97.2%		30	93.8%		40	100%		0.109
	< 10	2	2.8%		2	6.3%		0	-		
**Preoperative creatinine**				**1.19 (±.281)**			**1.29 (±.318)**			**1.11 (±.221)**	**0.009**
**(mg/dL)**	< 1.5	57	79.2%		21	65.6%		36	90%		0.011
	≥ 1.5	15	20.8%		11	34.4%		4	10%		
**Total operative time (minutes)**				384.65 (±79.5)			372.03 (±88.2)			394.75 (±71.3)	0.243
**Follow up period (months)**				38.58 (±22.8)			22.34 (±18.83)			51.58 (±16.6)	<.000
**RGP before RNU**	RGP	41	56.9%		26	81.3%		15	37.5%		<.000
	No RGP	31	43.1%		6	18.8%		25	62.5%		
**Time from RGP to RNU (days)**				**24.93 (±19.9)**			**22.81 (±17.93)**			**28.60 (±23.1)**	**0.411**
	< 7	13	31.7%		10	38.5%		3	20%		0.221
	≥ 7	28	68.3%		16	61.5%		12	80%		

### ultivariate analysis and the association between preoperative RGP and IVR

**Figure 1 –:**
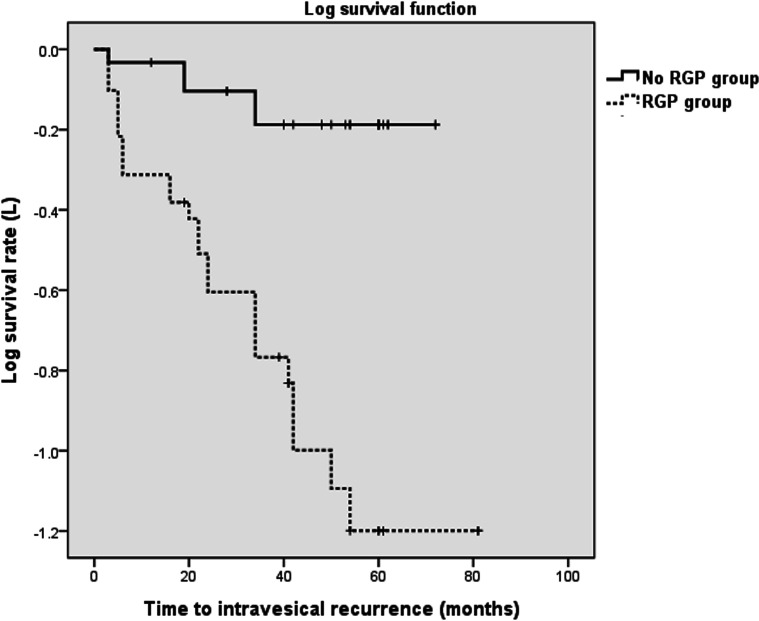
Kaplan-Meier curve for intravesical tumor recurrence after nephroureterectomy with or without preoperative RGP (p <0.001 from the log-rank test).

Multivariate Cox proportional hazard model analysis showed that tumor location (p=0.020, hazard ratio [HR]=2.742, 95% confidence index [CI]: 1.169-6.430), preoperative creatinine level (p=0.004, HR=6.351, 95% CI: 1.587-12.361), and receipt of preoperative RGP (p=0.045, HR=3.134, 95% CI: 1.027-9.560) were individually associated with IVR. Moreover, Kaplan-Meier analysis showed that preoperative RGP was significantly associated with IVR (p <0.001, Figure-1).

## DISCUSSION

The pathogenesis of IVR following RNU for UTUC remains unclear. Because IVR recurrence rates after RNU have been consistently reported to be clinically significant, several authors have sought to determine whether patient-, tumor-, and/ or treatment-specific parameters are associated with recurrence. Systemic reviews on RNU indicate previous bladder cancer, concomitant chronic kidney disease, tumor location on the ureter, the presence of LVI, tumor multiplicity, invasive pT stage, and positive surgical margins are associated with IVR in this patient population (6, 9). In a recently published meta-analysis, it was concluded that, in addition to these variables, receipt of URS before RNU elevated the risk of IVR (9), despite other evidence to the contrary (8, 11).

The present study is the first to report the effects of RGP before RNU on IVR, for patients that have not undergone preoperative URS. Advances in endoscopic equipment have made URS more accessible and have enabled its use in exploring the entire upper urinary tract. As a result, URS is rapidly replacing RGP for the identification of UTUC (12). Thus, our objective was not to evaluate the risk of IVR posed by preoperative RGP, but rather to investigate the mechanism responsible for IVR in this setting.

Several interesting observations were made during the present study. First, we detected a significant association between preoperative RGP and IVR following RNU. Importantly, the IVR rate was significantly higher in the RGP group than in the non-RGP group, and multivariate analysis adjusted for intergroup differences showed preoperative RGP, along with tumor location and preoperative creatinine level, independently predicted IVR. We believe this relationship supports the monoclonal theory of UTUC spread to the bladder, despite the fact that the retrospective design of this study prevented assessment of causality.

Second, we included intra-ureteral (or intra-pelvic) tumor size as a potential variable, because we considered a larger ureteral lesion would probably increase the risk of bladder exposure by facilitating tumor detachment during RGP. Two previous series have reported the effect of tumor size on IVR, but neither provided a clear definition of size or of the measuring method used. Ku et al. enrolled 181 patients that underwent RNU and divided them by tumor size using an approximate cut-off of 30mm but detected no association with IVR when applying Cox regression analysis (Odds ratio = 1.268, p=0.435) (13). Zou et al. studied 122 patients that underwent RNU with a mean tumor size of 29.9mm (range: 2-120mm), which was similar to that observed in the present study (mean = 34.9mm; range: 10-40mm). However, in this previous study (14), multivariate analysis failed to detect an effect of tumor size on IVR, which concurs with our findings and those of a recent meta-analysis (6). These observations imply that IVR is a complicated phenomenon with a mixed pathogenesis. In the present study, we observed that tumor location, rather than tumor size, had a significant influence on IVR.

Third, despite a strong relationship with preoperative RGP, the length of time between RGP and RNU was not observed to influence IVR. Originally, we considered the exposure duration for normal bladder urothelium to detach UTUC cells would be associated with IVR. However, the periods between RGP and RNU were similar in the IVR and non-IVR groups. Furthermore, when the RGP group was dichotomized using time from RGP to RNU cut-off periods of 7 days (22%), 10 days (31.7%), or the median period of 23 days (51.2%), no significant differences in IVR rates were observed (data not shown). Given that 73.2% of RNU procedures were performed within one month (97.6% within two months) of RGP, this finding implies an RGP to RNU time of longer than one month is required to influence IVR. In addition, given that a single session of URS plus RNU was not associated with an increase in IVR (15, 16), it would appear that members of the RGP group with almost a month interval had similar risks of developing IVR because all RGP procedures were performed separately.

We are aware that the present study has several limitations. First, because the contemporary diagnosis of UTUC was conducted based predominantly on CT and URS results rather than on RGP findings, we had to enroll patients over almost a decade, which raised issues regarding the effects of possible changes in UTUC management. For example, adjuvant chemotherapy on >pT3 disease was only conducted in 5 of 12 subjects, which may have biased IVR outcomes. In addition, the prolonged enrollment period, unfortunately, caused age to be significantly different in the RGP and non-RGP groups (Table-1). Second, postoperative follow-up after RNU was performed by four urological specialists; in addition, surgical volumes differed and indications for RGP were not standardized. Third, the study was performed using retrospective data collected at a single center, which cautions that the study outcomes should be interpreted with care. Fourth, the primary endpoint, IVR, was not determined by a single modality, but rather was determined by radiologic and cystoscopic evaluation regardless of bladder tumor status. Further study will no doubt shed additional light on the mechanisms associated with IVR and the effect of intra-ureteral instrumentation.

## CONCLUSIONS

Given the limitations of a retrospective, single-center series, RGP before RNU was shown to have a negative effect on IVR after surgery, regardless of the length of time between RGP and RNU. We suggest that the results provide evidence supporting the theory that monoclonal tumor cell spread underlies the pathogenesis of IVR.
